# Effects of Spirulina supplementation in patients with ulcerative colitis: a double-blind, placebo-controlled randomized trial

**DOI:** 10.1186/s12906-024-04400-w

**Published:** 2024-02-29

**Authors:** Sajjad Moradi, Reza Bagheri, Parsa Amirian, Mahsa Zarpoosh, Neda Cheraghloo, Alexei Wong, Mehdi Zobeiri, Mohammad Hassan Entezari

**Affiliations:** 1 Department of Nutrition and Food Sciences, Research Center for Evidence-Based Health Management, Maragheh University of Medical Sciences, Maragheh, Iran; 2 Department of Exercise Physiology, University of Isfahan, Isfahan, 8174673441 Iran; 3 General Practitioner, Kermanshah University of Medical Sciences (KUMS), Kermanshah, Iran; 4 Department of Epidemiology and Biostatistics, School of Public Health, Tehran University of Medical Sciences, Tehran, 1417613151 Iran; 5 Department of Health and Human Performance, Marymount University, Arlington, VA USA; 6 Department of Internal Medicine, School of Medicine, Kermanshah University of Medical Sciences, Kermanshah, Iran; 7 Department of Clinical Nutrition, School of Nutrition and Food Science, Isfahan University of Medical Sciences, Isfahan, Iran

**Keywords:** Spirulina, Ulcerative colitis, Health-related quality of life, Oxidative stress

## Abstract

**Aim:**

We conducted a randomized placebo-controlled trial to assess the efficacy of Spirulina (SP) supplementation on disease activity, health-related quality of life, antioxidant status, and serum pentraxin 3 (PTX-3) levels in patients with ulcerative colitis (UC).

**Methods:**

Eighty patients with UC were randomly assigned to consume either 1 g/day (two 500 mg capsules/day) of SP (*n* = 40) or control (*n* = 40) for 8 weeks. Dietary intakes, physical activity, disease activity, health-related quality of life, antioxidant status, erythrocyte sedimentation rate (ESR), and serum PTX-3 levels were assessed and compared between groups at baseline and post-intervention.

**Results:**

Seventy-three patients (91.3%) completed the trial. We observed increases in serum total antioxidant capacity levels in the SP supplementation group compared to the control group after 8 weeks of intervention (*p* ≤ 0.001). A within-group comparison indicated a trend towards a higher health-related quality of life score after 8 weeks of taking two different supplements, SP (*p* < 0.001) and PL (*p* = 0.012), respectively. However, there were no significant changes in participant’s disease activity score in response to SP administration (*p* > 0.05). Similarly, changes in ESR and PTX-3 levels were comparable between groups post-intervention (*p* > 0.05).

**Conclusions:**

SP improved antioxidant capacity status and health-related quality of life in patients with UC. Our findings suggest that SP supplementation may be effective as an adjuvant treatment for managing patients with UC. Larger trials with longer interventions periods are required to confirm our findings.

## Introduction

Ulcerative colitis (UC) is a prevalent type of Inflammatory Bowel Disease (IBD) characterized by chronic inflammation, ulcers in the distal part of the intestine, and clinically recurrent phases of aggravation and remission [1]. Although the etiology of UC is poorly elucidated, growing evidence has revealed that interactions between several components, including genetic variations in the intestinal microbiome, immune responses, and environmental factors, may play a role [2–5], UC represents an underlying cause of various other disorders, including intermittent diarrhea and constipation, cramping, abdominal, rectal, or joint pain, bleeding, and/or anemia [6–8]. Thus, identifying and treating pathophysiological factors can play an indispensable role in reducing UC-related complications.

Inflammatory markers, which involved the pathophysiology of UC, have received attention as a method for indirect assessment of UC and a potential therapeutic target for this condition [9]. To that end, acute phase inflammatory markers such as C reactive protein (CRP) or the erythrocyte sedimentation rate (ESR) in plasma are measured for this purpose [9, 10]. Emerging evidence also indicated that serum pentraxin-3 (PTX-3) is an important inflammatory marker for IBD [11, 12]. Short pentraxins (e.g., CRP, serum amyloid P) are produced by hepatocytes [13]; in contrast, PTX-3 as a long member of the pentraxin superfamily is produced by innate immunity cells in response to inflammatory cytokine and tissue damage [11, 12]. The PTX-3 is released mainly from neutrophils in inflamed colon tissue in UC patients, especially in crypt abscess injuries [14, 15]. Therefore, PTX-3 is an independent biomarker of disease activity produced at the site of inflammation, which may be helpful as a rapid disease activity biomarker to detect and treat primary local inflammation induced by epithelial damage and crypt abscess in patients with UC. Although immunosuppressants and anti-inflammatory medications are commonly used for patients with UC [16], these pharmacological treatments induce several adverse effects, including raised risk of infection, low bone mineral density, liver disease, tremor, eye disorders, gastrointestinal disease, pancreatitis, and antigen-antibody response [17, 18]. Thus, applying a comparably safer adjuvant treatment with fewer side effects and lower toxicities may be more favorable in UC management.

Over recent decades, evidence has supported the benefits of select herbal therapies on UC due to their bioactive compounds’ healing or antioxidant characteristics. These therapies are generally considered safe for managing UC-related complications [19–24]. Namely, Spirulina (SP; Arthrospira platensis) which is a biomass of cyanobacteria (blue-green algae) [25, 26]. This alga, predominantly classified as a phytomedicine, has been widely consumed as a dietary supplement or a whole food. It is considered a good source of essential nutrients, including phytochemicals (carotenoids, phycocyanins), minerals (calcium, iron), amino acids, essential fatty acids, vitamins (vitamin B12, provitamin A), and fiber [27–29]. SP has anti-inflammatory, antioxidant, liver-protecting, antiviral, and microbiome-regulating properties and has been suggested as an effective adjuvant therapy for managing many disorders [27, 28, 30, 31]. SP’s antioxidant and anti-inflammatory effects are specifically remarkable in the management of chronic diseases, including IBD [32, 33]. Previous studies documented that SP supplementation decreases inflammatory cytokines, such as tumor necrosis factor (TNF)-α [34], interleukin-6 (IL-6) [35], CRP [36], ESR [37], and PTX-3 [38]. In addition, several studies reported that SP administration significantly affects oxidant and antioxidant parameters. For instance, SP supplementation has been shown to reduce malondialdehyde (MDA) levels [39] and increase total antioxidant capacity (TAC), superoxide dismutase (SOD), and glutathione peroxidase (GPx) levels [35, 40–42]. However, these studies mainly focused on the effect of SP on inflammatory and antioxidant factors per se*,* and the effect of this microalgae compound has been poorly elucidated on clinical outcome measures of patients with inflammatory conditions, including those with chronic colitis. Therefore, the purpose of this investigation was to evaluate the effects of SP supplementation on disease activity, health-related quality of life, serum antioxidant status, and PTX-3 levels in patients with UC.

## Materials and methods

### Participants’ characteristics

Eighty patients with UC (age: 38.64 ± 11.30 years, height: 166 ± 8.57 cm, and BMI: 25.81 ± 4.96 kg/m^2^) referred to the Imam Reza Hospital (Kermanshah, Iran) were enrolled in the study. Participants were included if they (1) were clinically diagnosed with UC using a colonoscopy exam, clinical records, and pathology assessment; (2) were between 18 and 65 years of age; and (3) exhibited symptoms of active mild to moderate UC disease (5 ≤ or ≤ 12 scores based on the Simple Clinical Colitis Activity Index [SCCAI]) [43]. Patients were excluded if they (1) had good or severe ulcerative colitis (SCCAI scores of < 5 or > 12); (2) were pregnant or breastfeeding; (3) consumed antidepressants, anxiety medications, antioxidants, omega-3, or other supplements in the past 3 months; (4) smoked or consumed alcohol; (5) had heart, liver, kidney, cancer, thyroid, parathyroid, or other gastrointestinal conditions; or (6) had poor compliance to SR supplementation protocol (consumed < 90% of their supplements during the intervention period).

### Experimental protocol

This study was a double-blind and placebo-controlled randomized clinical trial. Prior to baseline measurements, participants were fully familiarized with all experimental procedures. Patients with active mild to moderate UC were randomly allocated to one of two groups: a SP supplementation (*n* = 40) or control (*n* = 40) utilizing simple randomization via a random number table. Prior to and following our eight-week study duration, measures of anthropometry, dietary intake, disease activity, health-related quality of life, serum antioxidant status, and PTX-3 levels were made. All patients were instructed to continue their usual lifestyles, including physical activity, dietary intake, and medication regimen throughout the study period. Compliance with the assigned intervention was evaluated through weekly phone calls and monitoring the number of supplement packages used. Patients, laboratory staff, researchers, and participants were blinded to the supplement allocation until the end of the trial period.

### Randomization and blinding

The study employed a simple randomization method, facilitated by a random number table. Throughout the duration of the trial, the participants, laboratory personnel, and researchers were blinded to treatment assignment. At no time during the intervention were the investigators and/or participants aware of which treatment was being provided to study participants.

### Spirulina supplementation

The SP group was supplemented with a 500 mg capsule of SP twice per day, before lunch and dinner. The control group received two placebo capsules containing 500 mg corn starch without chlorophyll during the same time periods. The placebo capsules had similar color, size, and shape compared to SP capsules. The selected dose and time of ingestion were based on prior investigations [44–48]. The SP powder was produced by the Javane Sabz Company, Shiraz, Iran. The chemical composition of Spirulina and placebo per 100 g is reported in Table 1. All chemical analytical procedures were completed in the Beh-Azma laboratory (Iran) in compliance with the assessment methods recommended by the Association of Analytical Communities. Both the SP and placebo capsules were prepared by researchers under sterile conditions, including the measurement of the weight and quality.

**Table 1:** Chemical composition of Spirulina and placebo per 1 g of product weight (capsule content)

Nutrients content	Spirulina	Placebo
Energy (kcal)	3.78	3.69
Carbohydrate (g)	0.15	0.89
Protein (g)	0.64	0.003
Fat (g)	0.08	0.0014
Fibre (g)	0.07	–
Ca (mg)	0.18	–
Fe (mg)	0.12	–
Zn (mg)	2.65	–
Mg (mg)	0.01	–
B6 (μg)	0.08	–
B9 (μg)	0.91	–
B12 (μg)	0.31	–
Phycocyanin(mg)	15	–
Chlorophyll (mg)	8	–
Beta-carotene (mg)	2.88	–
Moisture (%)	0.058	0.074
Total ash (%)	0.04	0.006
**Heavy metals and toxins**		
Lead (ppm)	0.0013	–
Arsenic (ppm)	0.0012	–
Mercury (ppm)	0.0001	–
Cadmium (ppm)	0.00008	–
Aflatoxin (ppb)	0.00043	–

### Outcome assessments

Height was evaluated via a nonelastic wall-mounted stadiometer, measured to the nearest 0.5 cm. Body mass was assessed with participants dressed in light clothes using a digital scale to the nearest 0.1 kg. Body mass index (BMI) was calculated using a formula: Weight (kg)/ the square of the body height (m^2^).

To assess the dietary intake of each patient, food diaries were collected for 3 days, including two weekdays and one weekend day. Nutrient intakes were assessed using the Nutritionist IV software (First Databank, San Bruno, CA) modified for Iranian foods. Physical activity levels were evaluated via a short form of the International Physical Activity Questionnaire (IPAQ) [49].

Patients’ disease activity was assessed using the SCCAI questionnaire score, which correlates closely to biochemical parameters of UC, and is valid and reliable for evaluating patients with this condition [50]. This questionnaire has various parts with a total score ranging from zero to 19. Higher scores indicate higher severity of UC symptoms during the past week. Moreover, the Short IBD Questionnaire (SIBDQ) score was used to assess health-related quality of life (HRQoL) in IBD patients. The validity and reliability of SIBDQ were first confirmed (r = 0.83) by Jowett et al. for patients with UC [51] and subsequently in an Iranian cohort [52]. Each question of SIBDQ has seven items and covers scores of 1 to 7.

Blood samples were obtained between 8:00, and 10:00 am after an overnight fast (12 hours) at baseline and post-intervention. The erythrocyte sedimentation rate (ESR) was determined by the Westergren assay. The blood samples were centrifuged (3500 rpm), and the aliquots were stored at − 80 °C before further analysis. Total antioxidant capacity (TAC) was assessed according to the ferric reducing antioxidant power (FRAP) method using a commercial kit (Kiazist, Iran). MDA level was evaluated based on the thiobarbituric acid reactive substance (TBARS) using a commercial kit (Kiazist, Iran). Superoxide dismutase (SOD) concentration was also measured according to the ability of Mn-SOD to inhibit the conversion of resazurin to resorufin accompanied by reducing superoxide radicals produced by the xanthine/xanthine oxidase system using a commercial kit (Kiazist, Iran). All intra- and inter-assay coefficients of variation were less than 10%. Finally, the serum PTX-3 levels were evaluated using the enzyme-linked immunosorbent assay (ELISA) with an intra- and inter-assay coefficient of variability (CV) less than 5% by a Human PTX-3 Test kit (Crystal, China) based on the manufacturer’s guideline.

### Sample size calculation

We conducted an a priori calculation of sample size using the G*Power analysis software [53], accounting for a Type I error rate of 5% and a statistical power of 80%. A minimum detectable effect size (i.e., Δ of clinical response) of 0.3 was considered clinically plausible using data from previous clinical trials in patients with UC [54, 55]. Our calculated sample size was 33 participants in each group. Ultimately, 40 patients in each experimental arm were estimated to be sufficient after considering a 20% dropout rate.

### Safety assessment

Adverse events that may or may not be associated with the study therapies, including abnormal gastrointestinal reactions, cardiovascular events, allergic reactions, and other medical conditions were recorded.

### Statistical methods

We presented continuous variables as mean ± standard deviation (SD) and expressed categorical variables as frequencies (percentage). The normality of data distribution was assessed using the Shapiro–Wilk test and Q–Q plot. For normally distributed variables, we used an independent sample t-test for comparison, and for non-normally distributed variables, the Mann–Whitney U test was employed. Categorical variables were analyzed using the chi-square test. The mean of markers between the SP and PL groups was compared using an independent t-test, and changes over time were evaluated utilizing a paired t-test. To examine the impact of the group on the markers in the post-test, controlling for the effects of baselines, we employed analysis of covariance (ANCOVA). If the homogeneity of variance was not met, we estimated the parameter with robust standard errors. The analyses were conducted using SPSS (version 26, Armonk, NY, USA) and STATA version 17 (Stata Corp LLC, TX, USA), and *p*-values less than 0.05 were considered statistically significant.

## Results

**Fig. 1:**
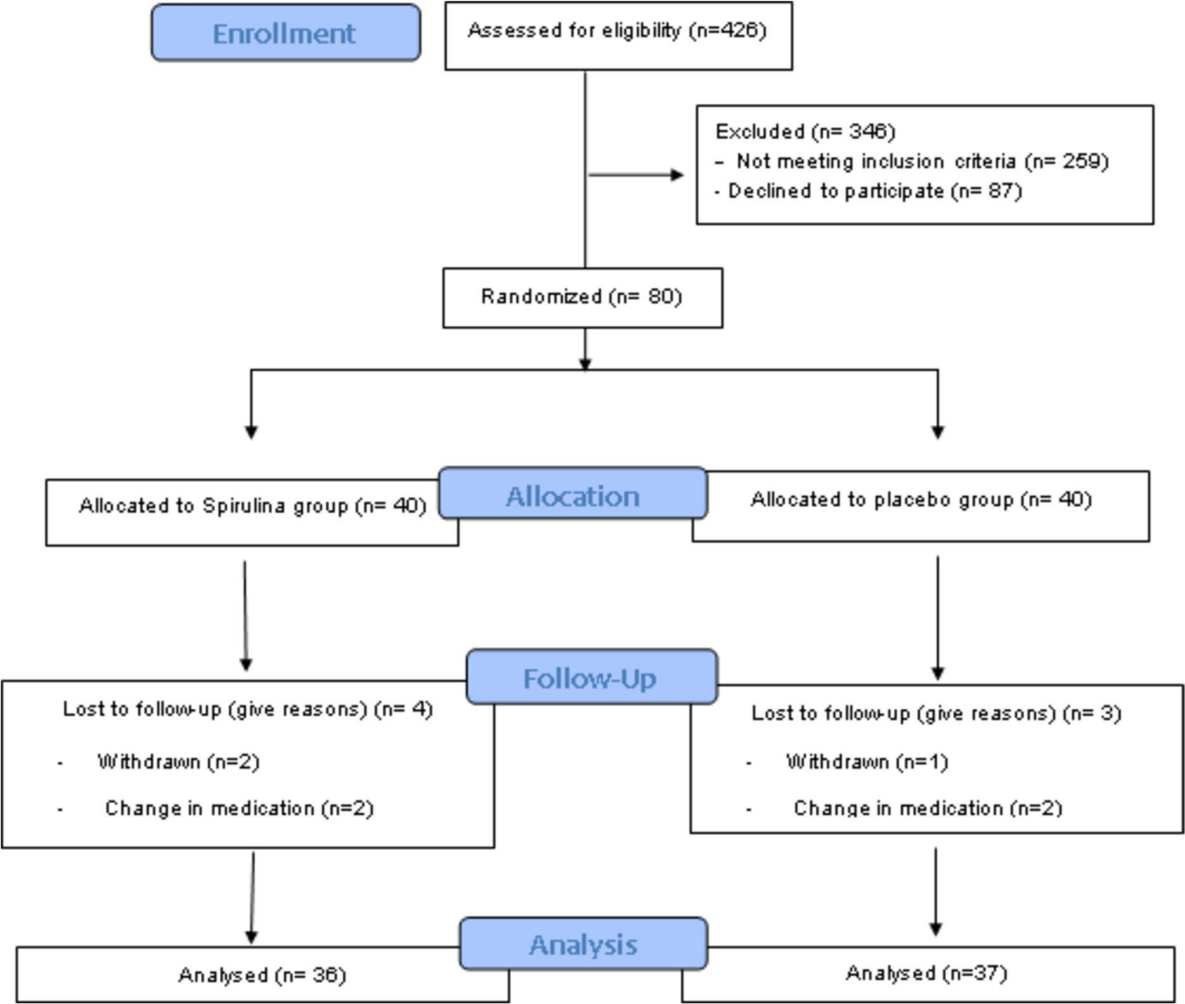
Participants’ flow diagram

Between May 2020 and January 2021, we screened 426 both female and male patients with UC. Of these, 346 were excluded for not meeting the exclusion criteria (*n* = 259) and for declining to participate (*n* = 87). Consequently, 80 participants with mild or moderate degrees of UC disease were enrolled in the current study and randomly assigned to the SP and control groups. Three patients withdrew before completing the study for personal reasons and four due to changes in their medication use (Fig. 1). Data are presented for the 73 patients (*n* = 36 and *n* = 37 in the SP and control groups, respectively) that completed our eight-week intervention. Participants’ compliance with their intervention was > 90% in both groups.

### Participant’s characteristics

The mean age of participants in SP and PL group was 37.80 and 39.50 years which was homogenate by the group. Results showed that the difference of markers between SP and PL was not significant in pretest (baseline) (Table 2). However, the mean of markers Calcium (*p* = 0.007, *ES*_Cohen ′ s d_ = 0.64), Magnesium (*p* = 0.040, *ES*_Cohen ′ s d_ = 0.49) and B9 (*p* = 0.012, *ES*_Cohen ′ s d_ = 0.60) in post-test were significantly different by group (Table 3). In addition, there was a significant mean difference in marker (post – pre) (SIBDQ score) in both groups (SP: *p* = < 0.001, *ES*_Cohen ′ s d_ = 0.87, PL: *p* = 0.012, *ES*_Cohen ′ s d_ =0.43). (Table 4).

**Table 2:** Participants’ baseline characteristics

Variables	Spirulina group (*n* = 36)	Placebo group (*n* = 37)	*p*
Age (years)	11.67 ± 37.77	39.48 ± 11.03	0.52 ^a^
Sex (female/male)	18/18	20/17	0.72^c^
Height (cm)	166.73 ± 8.38	165.37 ± 8.82	0.50 ^a^
Weight (kg)	72.33 ± 13.59	69.72 ± 14.54	0.43 ^a^
BMI (kg/m^2^)	26.01 ± 4.41	25.61 ± 5.05	0.73 ^a^
TAC (nmol/mL)	281.43 ± 53.35	259.92 ± 61.45	0.11^a^
MDA (nmol/mL)	60.74 ± 16.06	63.30 ± 26.36	0.61 ^a^
SOD (U/mL)	10.65 ± 4.08	10.21 ± 5.49	0.69 ^a^
ESR (mm/h)	20.61 ± 19.68	17.56 ± 13.64	0.44 ^a^
Pentraxin 3 (ng/mL)	13.49 ± 7.38	12.13 ± 8.33	0.37 ^a^
SCCAI score	8.16 ± 2.48	9.02 ± 2.58	0.15 ^a^
SIBDQ score	41.61 ± 10.95	42.08 ± 9.71	0.33 ^a^
Disease duration (year)	7.16 ± 5.59	5.21 ± 5.02	0.12 ^a^
Dose of Mesalazine (mg/day)	2277.77 ± 1614.41	1959.45 ± 1180.73	0.47^b^
Family history n (%)	8 (22.22)	7 (18.91)	0.72^c^
Current medication use n (%)			
Mesalazine (oral)	30 (83.33)	32 (86.48)	0.23^c^
Mesalazine (rectal)	11 (30.55)	12 (32.43)	0.32^c^
Sulfasalazine	6 (16.66)	3 (8.10)	0.69^c^
Prednisolone	3 (8.33)	3 (8.10)	0.35^c^
Azathioprine	6 (16.66)	5 (13.51)	0.62^c^

Variables are expressed as mean ± SD. *BMI* Body mass index, *TAC* total antioxidant capacity, *MDA* Malondialdehyde, *SOD* Superoxide dismutases, *ESR* Erythrocyte sedimentation rate, *SCCAI* Simple Clinical Colitis Activity Index

^a^*p* values resulted from independent t tests

^b^*p* values resulted from Mann–Whitney U test

^c^Chi-square for qualitative variables between the two groups

**Table 3:** Nutrients intake, physical activity, and anthropometrics at pre and post intervention

Variable	Group	Pre	Post	*P* value**
Energy (kcal/day)	SP	2138.9 ± 540.5	2170.5 ± 659.3	0.520
	PL	2104.9 ± 445.7	2118.4 ± 447.8	0.720
Carbohydrate (g/day)	SP	311.6 ± 105.1	308 ± 128.5	0.777
	PL	325.7 ± 113.7	397.2 ± 496.8	0.390
Protein (g/day)	SP	91.39 ± 31.84	91.2 ± 29.5	0.978
	PL	92.3 ± 25.1	102.8 ± 71	0.238
Fat (g/day)	SP	60.9 ± 19.7	67.6 ± 29.2	0.146
	PL	59.6 ± 20.2	60.6 ± 22.1	0.587
Saturated fats (g/day)	SP	19.4 ± 7.3	21.7 ± 11.4	0.223
	PL	20.5 ± 5.9	21.1 ± 7.1	0.370
Cholesterol (mg/day)	SP	406.1 ± 295	400.6 ± 285.4	0.893
	PL	365.7 ± 188.8	380.9 ± 213.3	0.450
Linolenic fat (g/day)	SP	0.35 ± 0.32	13.3 ± 54.4	0.162
	PL	0.35 ± 0.28	31.9 ± 76.3	0.016**
Linoleic fat (g/day)	SP	7.7 ± 4.5	32.1 ± 80.6	0.077
	PL	7.8 ± 4.2	55.3 ± 112.9	0.014**
Eicosapentaenoic acid (g/day)	SP	0.04 ± 0.1	0.04 ± 0.11	0.949
	PL	0.03 ± 0.1	0.06 ± 0.13	0.165
Docosahexaenoic acid (g/day)	SP	0.07 ± 0.16	37.7 ± 225.6	0.323
	PL	0.07 ± 0.1	0.1 ± 0.3	0.104
Polyunsaturated fats (g/day)	SP	15.1 ± 10.9	17.1 ± 11.1	0.344
	PL	12.3 ± 8.1	12.3 ± 7.6	0.928
Monounsaturated fats (g/day)	SP	19.5 ± 8	21.3 ± 10.6	0.184
	PL	17.4 ± 6.8	18.4 ± 7.6	0.137
Dietary fiber (g/day)	SP	15.6 ± 7	16 ± 8.1	0.698
	PL	17.9 ± 17.5	19 ± 17.7	0.206
Arginine (mg/day)	SP	433.87 ± 492.11	402.1 ± 569.6	0.600
	PL	401.1 ± 391.4	527.3 ± 523.7	0.047**
Alanine (mg/day)	SP	401.3 ± 484.2	385.1 ± 564.1	0.734
	PL	400 ± 390.3	524.6 ± 510.7	0.052
Glutamic Acid(mg/day)	SP	2064.4 ± 1770.6	1920.9 ± 2055.2	0.546
	PL	1890.6 ± 1541.6	2458.6 ± 1966.8	0.011
Leucine (mg/day)	SP	5526.9 ± 1970.3	5361.9 ± 2220.4	0.626
	PL	5388 ± 1407.3	5911.5 ± 1764.7	0.047**
Methionine (mg/day)	SP	1764.7 ± 704.8	1718.8 ± 734.3	0.701
	PL	1705.2 ± 472.8	1812.1 ± 580.3	0.172
Calcium (mg/day)	SP	814.4 ± 269.7	697.4 ± 295.8*	0.003**
	PL	891.7 ± 415.1	930.4 ± 417.1	0.181
Phosphorus (mg/day)	SP	1160.5 ± 416.8	1189.6 ± 493.9	0.682
	PL	1202.3 ± 367.2	1279.5 ± 347.5	0.087
Iron (mg/day)	SP	16.48 ± 5.58	17.2 ± 6.6	0.330
	PL	18.8 ± 5.3	21.6 ± 20.2	0.365
Copper (mg/day)	SP	1.1 ± 0.5	1.2 ± 0.6	0.245
	PL	1.1 ± 0.4	1.2 ± 0.4	0.157
Magnesium (mg/day)	SP	208.6 ± 83.1	213.8 ± 84.2*	0.627
	PL	238.9 ± 64.9	252.7 ± 74.6	0.082
Zinc (mg/day)	SP	8.77 ± 2.7	9.6 ± 3.4	0.193
	PL	9.8 ± 2.5	10.1 ± 2.7	0.334
Selenium (mg/day)	SP	0.11 ± 0.05	0.1 ± 0.05	0.220
	PL	0.09 ± 0.04	0.1 ± 0.06	0.037**
B6 (mg/day)	SP	1.3 ± 0.5	1.4 ± 0.6	0.562
	PL	1.6 ± 0.9	1.5 ± 0.5	0.283
B9 (Ug/day)	SP	301 ± 150	237.6 ± 118.7*	0.005**
	PL	311.5 ± 128.	319.4 ± 152.1	0.570
B12 (Ug/day)	SP	4.5 ± 2.3	4.4 ± 2.3	0.736
	PL	4.8 ± 2.3	4.6 ± 1.6	0.495
Vitamin C (mg/day)	SP	152.7 ± 72.1	102.3 ± 82	< 0.001**
	PL	143.4 ± 109.2	148.8 ± 122.8	0.573
Vitamin E (mg/day)	SP	2.7 ± 1.8	1.9 ± 1	0.014**
	PL	2.5 ± 0.8	2.3 ± 1.1	0.435
Lutein (mg/day)	SP	890.5 ± 524.6	772.3 ± 436	0.349
	PL	804.8 ± 447.9	868.1 ± 559.9	0.539
Lycopene (mg/day)	SP	1505.7 ± 1124.9	1481 ± 1148.3	0.421
	PL	1543.4 ± 1993.3	1518.2 ± 2001.9	0.461
α–Carotene (mg/day)	SP	1815.6 ± 1436.2	1737.1 ± 1474.6	0.186
	PL	1878.2 ± 980.4	1755.3 ± 1059.3	0.197
β-Carotene (mg/day)	SP	207.1 ± 124.7	238.5 ± 156	0.085
	PL	217.7 ± 62.9	263.3 ± 112.4	0.004**
β-Cryptoxanthin (mg/day)	SP	90.4 ± 78.2	74.8 ± 72.5	0.167
	PL	93.5 ± 75.8	101 ± 75.8	< 0.001**
α-Tocopherol (mg/day)	SP	4.4 ± 1.3	4.6 ± 1.1	0.073
	PL	4.1 ± 1.3	4.4 ± 1.2	0.010**
Physical activity level (MET/h/day)	SP	24.6 ± 1.6	24.64 ± 1.7	0.955
	PL	23.9 ± 1.9	23.8 ± 1.9	0.413
Wieght (kg)	SP	72.3 ± 13.5	72.8 ± 13.9	0.028**
	PL	69.7 ± 14.5	70 ± 14.4	0.156
BMI (kg.m^−2^)	SP	26 ± 4.4	26.2 ± 4.5	0.021**
	PL	25.6 ± 5.5	25.7 ± 5.5	0.141
WHR	SP	0.91 ± 0.07	0.91 ± 0.07	0.339
	PL	0.91 ± 0.09	0.91 ± 0.09	0.416

*BMI* Body mass index, *WHR* Waist to hip ratio

*The *p*-value of independent t-test

**The *p*-value of paired t-test

**Table 4:** Descriptive statistics of variables by group and time-point

Variable	Group	Post - Pre	*P*-value*
SOD (U/mL)	SP	0.24 ± 5.45	0.795
	PL	− 0.35 ± 6.62	0.752
TAC (nmol/mL)	SP	19.18 ± 65.83	0.089
	PL	−11.33 ± 61.92	0.273
Pentraxin 3 (ng/mL)	SP	−2.03 ± 7.12	0.096
	PL	−0.23 ± 5.60	0.799
MDA (nmol/mL)	SP	−7.42 ± 16.45	0.010
	PL	−2.11 ± 17.61	0.471
ESR (mm/h)	SP	−4.61 ± 19.01	0.155
	PL	−0.86 ± 9.38	0.578
SCCAI score	SP	−0.42 ± 3.56	0.487
	PL	−0.24 ± 3.76	0.696
SIBDQ score	SP	6.69 ± 7.70	< 0.001
	PL	2.89 ± 6.67	0.012

*TAC* total antioxidant capacity, *MDA* Malondialdehyde, *SOD* Superoxide dismutase, *ESR* Erythrocyte sedimentation rate, *SCCAI* Simple Clinical Colitis Activity Index, *SP* Spirulina, *PL* Placebo

*The *p*-value of paired t-test

In the next step, the ANCOVA was used to evaluate the impact of the group on markers measured in post-test adjusting the baselines. Accordingly, the post means of protein (β = 1.22, 95% CI (0.87, 1.60), *ES*_*Partial Eta Squared*_ = 0.41) and Iron (β = 1.16, 95% CI (0.56, 1.76), *ES*_*Partial Eta Squared*_ =0.17) were significantly higher in SP compared to PL. However, the waist-to-height ratio (β = 1.00, 95% CI (0.95,1.05), *ES*_*Partial Eta Squared*_ = 0.95), BMI (β = 1.00, 95% CI (0.99, 1.04), *ES*_*Partial Eta Squared*_ = 0.99), physical activity (β = 1.00, 95% CI (0.99,1.01), *ES*_*Partial Eta Squared*_ = 0.99), energy (β = 1.00, 95% CI (0.88,1.13), *ES*_*Partial Eta Squared*_ = 0.78), vitamin B1 (β = 1.00, 95% CI (0.96,1.04), *ES*_*Partial Eta Squared*_ = 0.97), Lycopene (β = 1.00, 95% CI (0.97, 1.03), *ES*_*Partial Eta Squared*_ = 0.98), had equal mean value in SP and PL group. (Table 5).

**Table 5:** The impact of SP vs. PL on the markers

Variables	Contrast	β (SE)	95% CI	*P*-value
ESR (mm/h)	SP vs. PL	0.42(0.08)^**#**^	0.26,0.58	< 0.001
SOD (U/mL)	SP vs. PL	0.12(0.11)	−0.09,0.34	0.252
MDA (nmol/mL)	SP vs. PL	0.60(0.08)	0.44,0.76	< 0.001
TAC (nmol/mL)	SP vs. PL	0.83(0.13)	0.60,1.10	< 0.001
Pentraxin 3 (ng/mL)	SP vs. PL	0.60(0.08)	0.42,0.75	< 0.001
Energy (kcal/day)	SP vs. PL	1.00(0.06)	0.88,1.13	< 0.001
Protein (g/day)	SP vs. PL	1.22(0.17)	0.87,1.60	< 0.001
Fat (g/day)	SP vs. PL	0.82(0.11)	0.60,1.03	< 0.001
Saturated fats (g/day)	SP vs. PL	0.74(0.15)	0.45,1.03	< 0.001
Polyunsaturated fats (g/day)	SP vs. PL	0.53(0.10)	0.33, 0.73	< 0.001
Linoleic fat (g/day)	SP vs. PL	2.50(2.70)	−2.86, 7.82,5.90	0.357
Docosahexaenoic acid (g/day)	SP vs. PL	−66.55 (129.57)	− 324.98, 191.88	0.609
Sodium (g/day)	SP vs. PL	0.85(0.092)	0.66,1.03	< 0.001
Iron (mg/day)	SP vs. PL	1.16(0.30)	0.56,1.76	< 0.001
Magnesium (mg/day)	SP vs. PL	0.79(0.09)	0.61,0.96	< 0.001
Zinc (mg/day)	SP vs. PL	0.54(0.13)	0.29, 0.79	< 0.001
Manganese (mg/day)	SP vs. PL	0.54(0.08)	0.38,0.70	< 0.001
Fluoride (mg/day)	SP vs. PL	0.90(0.07)	0.76,1.03	< 0.001
Vitamin A (IU/day)	SP vs. PL	0.70(0.09)	0.49,0.86	< 0.001
Vitamin E (mg/day)	SP vs. PL	0.24(0.09)	0.06,0.42	0.008
B1(mg/day)	SP vs. PL	1.00(0.021)	0.96,1.04	< 0.001
B3 (mg/day)	SP vs. PL	0.90(0.04)	0.80,0.98	< 0.001
B9 (Ug/day)	SP vs. PL	0.68(0.08)	0.51,0.85	< 0.001
B5 (mg/day)	SP vs. PL	0.59(0.13)	0.33,0.86	< 0.001
Vitamin C (mg/day)	SP vs. PL	0.97(0.07)	0.84,1.11	< 0.001
Vitamin K (mg/day)	SP vs. PL	0.74(0.07)	0.60,0.88	< 0.001
Soluble fiber (g/day)	SP vs. PL	0.85(0.08)	0.70,1.00	< 0.001
Crude fiber (g/day)	SP vs. PL	0.76(0.09)	0.57, 0.95	< 0.001
Glucose (g/day)	SP vs. PL	0.65(0.07)	0.50,0.80	< 0.001
Fructose (g/day)	SP vs. PL	0.64(0.08)	0.49,0.80	< 0.001
Lactose (g/day)	SP vs. PL	0.61(0.10)	0.40, 0.82	< 0.001
Tryptophan(mg/day)	SP vs. PL	0.60(0.11)	0.35,0.80	< 0.001
Isoleucine (mg/day)	SP vs. PL	0.54(0.11)	0.32,0.76	< 0.001
Lysine (mg/day)	SP vs. PL	0.46(0.10)	0.25,0.67	< 0.001
Cystine (mg/day)	SP vs. PL	0.65(0.11)	0.44,0.86	< 0.001
Tyrosine (mg/day)	SP vs. PL	0.57(0.11)	0.35,0.78	< 0.001
Arginine (mg/day)	SP vs. PL	0.78(0.12)	0.55, 1.01	< 0.001
Alanine (mg/day)	SP vs. PL	0.77 (0.12)	0.54,1.00	< 0.001
Glutamine (mg/day)	SP vs. PL	0.75(0.12)	0.51,0.98	< 0.001
Proline (mg/day)	SP vs. PL	0.68(0.10)	0.47,0.88	< 0.001
Ash (g/day)	SP vs. PL	0.002(0.08)	−0.16,0.16	**0.976**
Carbohydrate (g/day)	SP vs. PL	0.66(0.39)	−0.12, 1.44	0.097
Cholesterol (mg/day)	SP vs. PL	0.72(0.09)	0.55, 0.89	< 0.001
Monounsaturated fats (g/day)	SP vs. PL	0.93(0.09)	0.74, 1.13	< 0.001
Oleic fat (g/day)	SP vs. PL	0.03(0.03)	−0.02,0.09	0.238
Linolenic fat (g/day)	SP vs. PL	62.18 (25.30)	11.72, 112.65	−0.016
Docosahexaenoic acid (g/day)	SP vs. PL	−66.55(129.57)	−324.98,191.88	0.609
Potassium (mg/day)	SP vs. PL	0.73(0.10)	0.53,0.92	< 0.001
Calcium (mg/day)	SP vs. PL	0.87(0.07)	0.74,1.00	< 0.001
Phosphorus (mg/day)	SP vs. PL	0.68(0.10)	0.48, 0.88	< 0.001
Copper (mg/day)	SP vs. PL	0.50(0.11)	0.27, 0.72	< 0.001
Selenium (mg/day)	SP vs. PL	0.50(0.12)	0.25,0.74	< 0.001
Chromium(mg/day)	SP vs. PL	−0.002(0.10)	−0.19, 0.19	0.986
Molybdenum (mg/day)	SP vs. PL	0.69(0.12)	0.45, 0.93	< 0.001
B 2 (mg/day	SP vs. PL	0.80(0.08)	0.65,0.95	< 0.001
B 6 (mg/day)	SP vs. PL	0.36(0.08)	0.19, 0.53	< 0.001
B12 (Ug/day)	SP vs. PL	0.50(0.08)	0.33,0.67	< 0.001
Biotin (mg/day)	SP vs. PL	0.73(0.07)	0.59,0.87	< 0.001
Vitamin D (*μg/d*)	SP vs. PL	0.01(0.06)	−0.11, 0.13	0.827
Dietary fiber (g/day)	SP vs. PL	0.94(0.05)	0.84,1.04	< 0.001
Insoluble fiber (mg/day)	SP vs. PL	0.06(0.04)	−0.03,0.14	0.166
Sugar (g/day)	SP vs. PL	0.77(0.08)	0.60,0.93	< 0.001
Galactose (g/day)	SP vs. PL	0.001(0.051)	−.10,0.10	0.990
Sucrose (g/day)	SP vs. PL	0.73(0.10)	0.52,0.93	< 0.001
Maltose (g/day)	SP vs. PL	0.00(0.03)	−0.05, 0.05	0.993
Threonine (mg/day)	SP vs. PL	0.43(0.10)	0.24,0.62	< 0.001
Leucine (mg/day)	SP vs. PL	0.63(0.12)	0.40,0.87	< 0.001
Methionine (mg/day)	SP vs. PL	0.61(0.11)	0.40,0.83	< 0.001
Phenylalanine (mg/day)	SP vs. PL	0.61(0.11)	0.39,0.82	< 0.001
Valine (mg/day)	SP vs. PL	0.62(0.11)	0.39,0.84	< 0.001
Histidine (mg/day)	SP vs. PL	0.52(0.11)	0.30,0.74	< 0.001
Aspartate (mg/day)	SP vs. PL	0.46(0.11)	0.24,0.68	< 0.001
Glycine (mg/day)	SP vs. PL	0.48(0.11)	0.26,0.69	< 0.001
Serine (mg/day)	SP vs. PL	0.48(0.11)	0.26,0.69	< 0.001
Lutein (mg/day)	SP vs. PL	0.03(0.12)	−0.22, 0.28	0.820
Lycopene (mg/day)	SP vs. PL	1.00(0.014)	0.97,0.103	< 0.001
α-Carotene (mg/day)	SP vs. PL	0.97(0.05)	0.88,1.06	< 0.001
β-Carotene (mg/day)	SP vs. PL	0.94(0.12)	0.70,1.18	< 0.001
β-Cryptoxanthin (mg/day)	SP vs. PL	0.78(0.07)	0.65, 0.91	< 0.001
α-Tocopherol (mg/day)	SP vs. PL	0.81(0.04)	0.73,0.90	< 0.001

# Parameter estimates with robust standard errors based on the original asymptotic or large sample robust, empirical, or “sandwich” estimator of the covariance matrix of the parameter estimates

*TAC* total antioxidant capacity, *MDA* Malondialdehyde, *SOD* Superoxide dismutase, *ESR* Erythrocyte sedimentation rate, *SCCAI* Simple Clinical Colitis Activity Index, *SP* Spirulina, *PL* Placebo

### Dietary intake and physical activity levels

As Table 3 shows, most nutrients remained unchanged over time except for calcium, magnesium, and vitamin B9 in SP group. In addition, the independent t-test (Table 3) indicated that the levels of all the nutrients were significantly different in the SP compared to the PL group, with the exception of linoleic acid, oleic acid, docosahexaenoic acid, α-Tocopherol, vitamin B6, vitamin D, fiber, galactose, and maltose and lutein (*p* > 0.05). Physical activity **(**Table 3**)** remained unchanged over time in both groups (*p* > 0.05).

### Health-related quality of life and SCCAI score

**Fig. 2:**
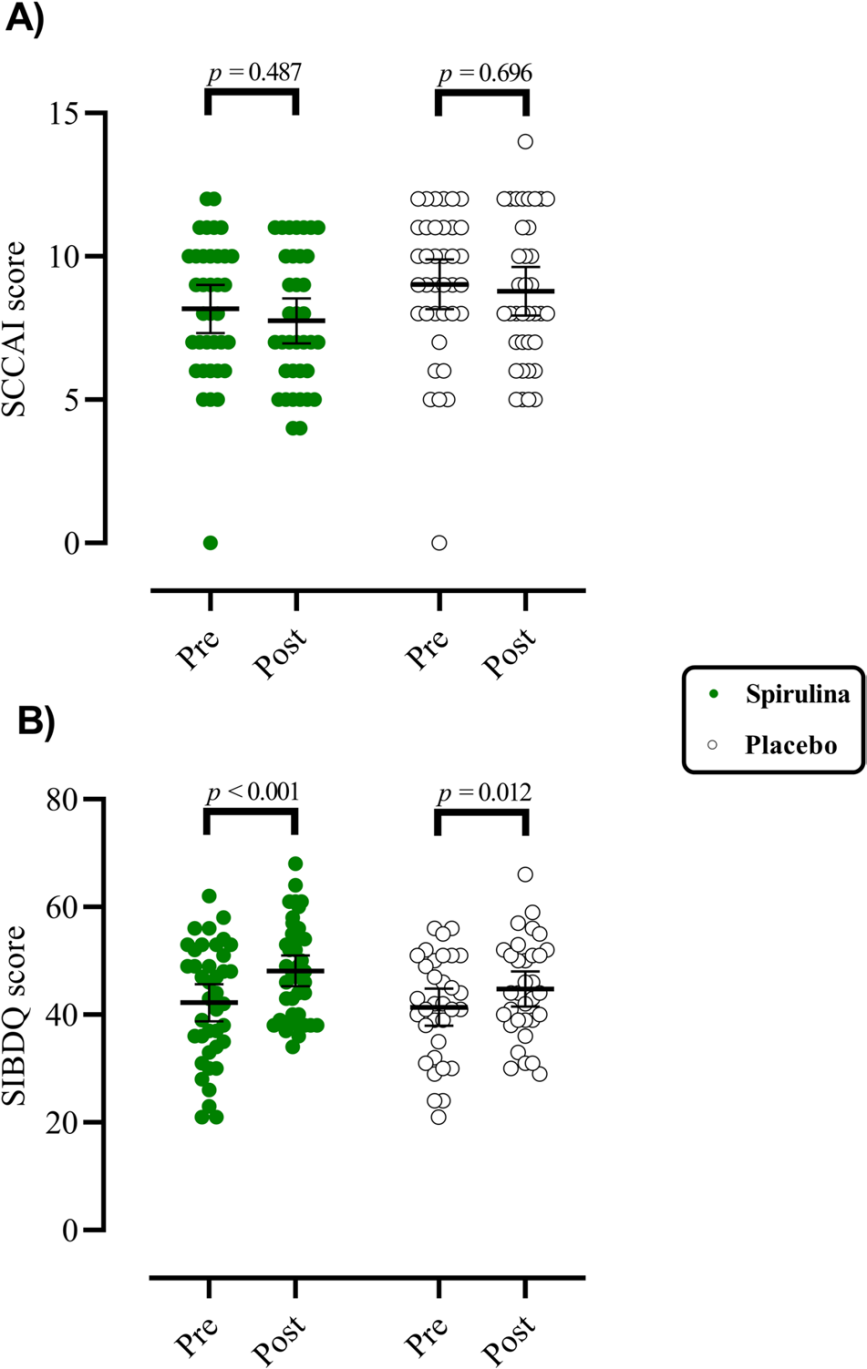
The effects of spirulina supplementation on SIQBD and SCCAI scores in patients with ulcerative colitis

The effect of SP administration on SIQBD and SCCAI scores in patients with UC is reported in Fig. 2**.** A within-group comparison indicated a trend towards a higher SIQBD score after 8 weeks of taking two different supplements, SP and PL, respectively (*p* < 0.001, *ES*_Cohen ′ s d_ = 0.87 and *p* = 0.012, *ES*_Cohen ′ s d_ = 0.43). However, there were no significant changes in participants SCCAI score in response to SP administration (*p* > 0.05).

### Antioxidant status and inflammatory markers

**Fig. 3:**
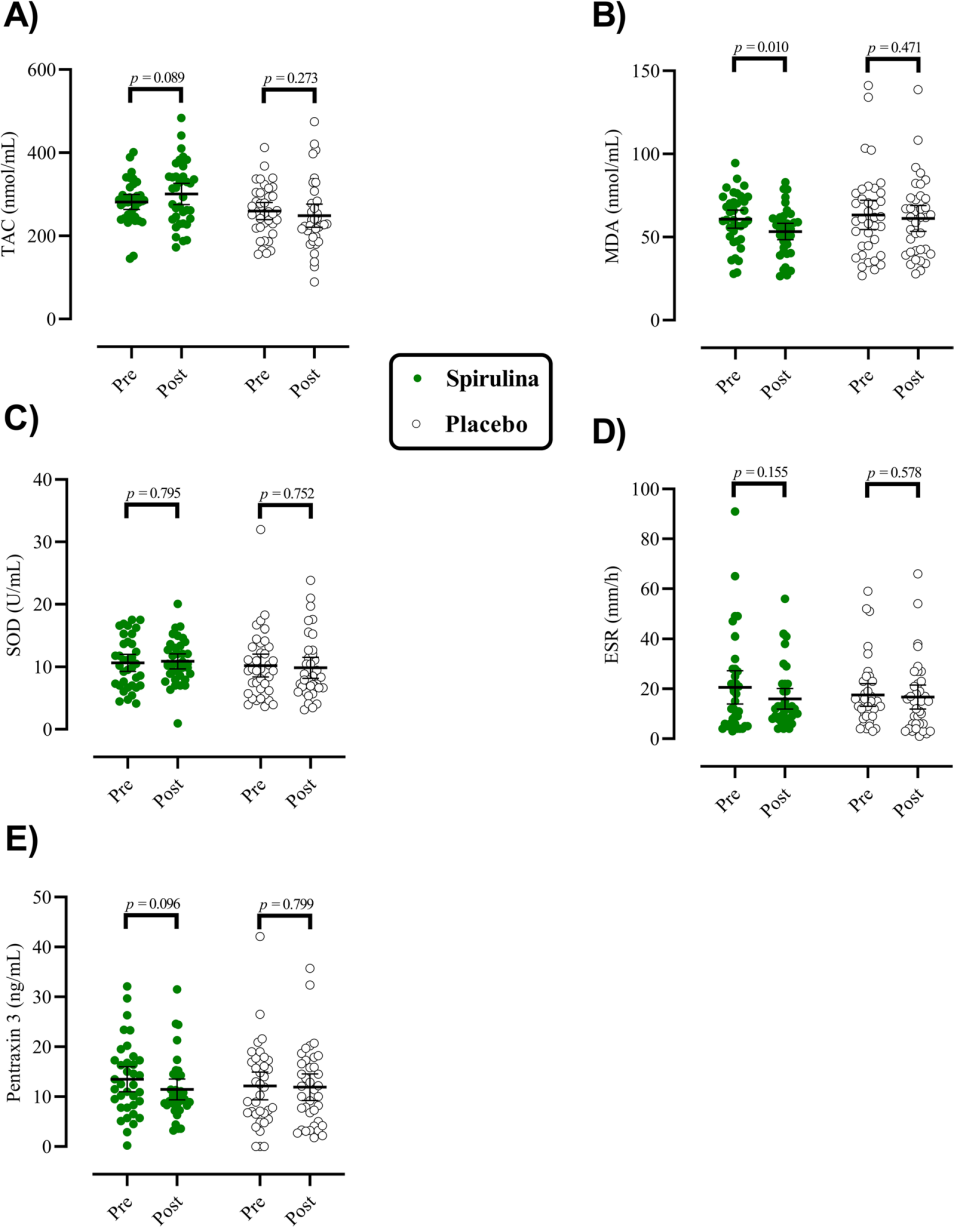
The effects of spirulina supplementation on antioxidant status and inflammatory markers

The effect of SP supplementation on antioxidant status and inflammatory parameters in patients with UC is reported in Fig. 3**.** The within-group comparison revealed a significant decrease in serum MDA after 8 weeks of SP supplementation (*p* = 0.01, *ES*_Cohen ′ s d_ = 0.45), but their TAC, SOD, ESR, and PTX-3 remained unchanged post-intervention (*p* > 0.05). In contrast, the within-group comparison revealed no significant changes in serum antioxidant status and inflammatory parameters in the control group (*p* > 0.05). Our ANCOVA analyses revealed a significant increase in serum TAC after 8 weeks of intervention in the SP supplementation group vs. the control group (β = 0.83, 95% CI (0.60,1.10), *ES*_*Partial Eta Squared*_ = 0.37). Moreover, no significant differences were observed in changes of MDA, SOD, ESR, and PTX-3 levels between groups from baseline to post-intervention (*p* > 0.05).

### Adverse effects

Overall, SP supplementation was well tolerated by patients and did not yield any severe adverse effects (e.g., allergic reactions). However, 5 out of 36 patients (13.8%) reported mild adverse effects, evidenced by mild bloating early in the trial. All bloating events were resolved later during the intervention.

## Discussion

The present trial evaluated the efficacy of SP supplementation on disease activity, health-related quality of life, serum antioxidant status, and PTX-3 levels in patients with UC. Our outcomes showed that; (1) SP supplementation improved serum TAC levels and stool frequency compared to the control group, (2) there were between-group significant differences in changes of health-related quality of life score, and (3) changes in ESR were comparable between groups.

Our observations indicate that SP supplementation may enhance antioxidant capacity and ameliorate oxidation status in patients with UC. Our observations corroborate the antioxidant properties of SP reported in recent studies [56]. Coskun et al. [57] and Abdel-Daim et al. [58] reported that SP intake led to a considerable enhancement of antioxidant potential and consequently reduced lipid peroxidation in rat models with acid-induced colitis. Similarly, Szulinska et al. [35] showed that 2 g/d of SP supplementation for a period of 3 months significantly promoted total antioxidant status in obese participants. Ismail et al. [59] also revealed that 1 g/d of SP administration for 2 months significantly reduced serum content of lipid peroxidation products and improved the antioxidant-related activity of enzymes, such as SOD and glutathione-s-transferase (GST) in patients with chronic obstructive pulmonary disease (COPD). Two unique pigments, the blue C-phycocyanin and yellow-to-red carotenoids are the most critical bioactivities influencing the antioxidant properties of SP [56, 60]. The SP-derived C-phycocyanin can have health-protective properties against oxidative stress harms through scavenging reactive oxygen species (ROS) and decreasing lipid peroxidation in liver microsomes [56, 60, 61]. Moreover, the yellow-to-red carotenoid content of SP acts as an antioxidant by decreasing oxygen-mediated lipid peroxidation and inhibiting the intracellular accumulation of ROS [56, 60, 62]. Accordingly, SP may alleviate UC symptoms by inhibiting oxidative stress and associated complications.

Another important finding from the current work is an improved health-related quality of life score in patients who received the SP supplementation compared to controls. An improved stool frequency score is among the most critical factors in improving the quality of life in patients with UC [63]. Besides pharmaceutical agents, various complementary therapies have been proposed to improve stool frequency and disease activity and increase the quality of life in patients with colitis [19, 64–66]. Of current therapies, the modification of the intestine microbiome by probiotics or symbiotics has received significant attention [64, 65, 67, 68]. Studies have documented that combination therapies containing *Lactobacillus* and *Bifidobacterium* strains and mesalazine decreased stool frequency and extended remission periods in patients with UC [67, 69]. Also, SP has prebiotic effects and can modulate gut microbiota [30, 70, 71]. In a dose-response model, Hu et al. [70] reported that oral SP supplementation altered the colonic microbial community in healthy mice. Similarly, Neyrinck et al. [30] reported that mice models supplemented with 5% SP had enhanced gut microbiota, especially regarding *Roseburia* and *Lactobacillus* strains. Further, extracellular products of SP significantly improve the growth and survival of the *Lactobacillus* and *Bifidobacterium* strains [71, 72]. The SP gut microbial modulating properties have been attributed to its high levels of phenolic bioactive, free amino acids, and nitrogenous compounds [71, 73].

Another mechanism by which SP affects intestinal health may be related to regulating adipokines [74–76]. In vitro, in vivo, as well as human models have proposed an interconnected and complex role for leptin, ghrelin, and resistin as pivotal mediators of pro-inflammation that may trigger UC [75, 77, 78]. In a mouse model study, Fujimoto et al. [79] reported that SP was associated with significantly lower leptin concentration. Likewise, Heo et al. [80] reported that SP administration attenuated leptin levels and other metabolites in serum. Moreover, Akbarpour et al. [81] showed a significant decrease in serum resistin concentrations following SP administration in patients with type 2 diabetes. This evidence suggests that the adipokine-regulating benefits of SP supplementation may play a role in managing patients with UC.

The SP has antiangiogenesis properties and fosters wound healing and health status in patients with UC [82, 83]. Angiogenesis is required to supply oxygen and nutrients to healing regions and is essential for lesion healing and tissue regeneration. However, angiogenesis attracts more inflammatory cells and cytokines, aggravating pro-inflammation in a vicious circle that exacerbates mucosal injury in patients with UC [84–86]. A critical factor in regulating the angiogenesis process in UC is the vascular endothelial growth factor (VEGF), which can be targeted for complementary treatment and ameliorating pathologic angiogenesis [84, 85]. To that end, Mahmoud et al. [82] have revealed SP antiangiogenic effects mediated by modulated VEGF expression in human colorectal carcinomas (HCC) -bearing mice. Similarly, Aldina et al. [87] showed antiangiogenic impacts of SP through decreasing VEGF expression in the cornea inflammation model in rats. However, other studies conducted by Zeinalian et al. [88] and Mehdinezhad et al. [89] demonstrated that SP did not alter serum VEGF in participants with obesity or diabetic rats. Due to the contradictory results in the available literature, further clinical trials are required to elucidate the potential role of angiogenesis and VEGF modulation in the improvement of disease activity and gut health after SP supplementation in patients with UC.

Several studies have suggested that using acute phase inflammatory markers, such as ESR and PTX-3 are non-invasive, safe, and available methods to reflect disease activity in patients with IBD [9, 11, 12, 15]. Our work revealed marginally significant changes in PTX-3 levels and non-significant changes in ESR after 8 weeks of SP administration compared to the control group. The PTX-3 is the longest member of the pentraxins family released mainly from neutrophils in inflamed colon tissue, especially in crypt abscess injuries of patients with UC [14]. The PTX-3-expressing cells and inflamed neutrophils have been shown to elevate proinflammatory reactions in the colon [14, 90]. Our study was conducted on patients with mild or moderate severity of UC who consumed anti-inflammatory medications, such as mesalazine, to attenuate pro-inflammation in the colon tissues [91]. These medications are known to modulate inflammatory reactions and their systemic biomarkers in patients with UC. Therefore, we may not have captured any net anti-inflammatory effect of SP supplementation by conducting a routine inflammatory profile test. Importantly, we are the first study to show the potential beneficial effect of SP supplementation on PTX-3 levels in individuals with UC.

## Strengths and limitations

Among our strengths is that this is the first trial to examine the efficacy of SP supplementation in health status among free-living patients with UC. Furthermore, most participants who completed the trial indicated good compliance with their therapy. Additional strengths of this work include a homogeneous cohort with UC and accounting for patients’ physical activity and dietary intake levels during the intervention as critical confounding factors.

Our study had some limitations. We did not perform colonoscopy or tissue biopsy to evaluate the severity of UC disease due to the invasive nature of these procedures, which might have led to higher attrition rates. However, we applied a valid and reliable SCCAI questionnaire as an effective tool to assess disease status. Another limitation is our use of a per-protocol analysis, which is susceptible to confounding biases, making its findings less generalizable to the broader patient population than those obtained from intention-to-treat analysis. We also did not measure specific disease-related biochemical markers, such as C-reactive protein or fecal calprotectin, which would have strengthen our study design as these markers have better correlation with endoscopic activity in UC. Furthermore, this study did not evaluate the dose-dependent efficacy of SP supplementation; therefore, these relationships remain to be elucidated in future work. Additionally, the intervention period in the current trial was likely not long enough to elicit drastic clinical changes. Consequently, longer interventions utilizing SP supplementation in patients with UC are warranted. Other important components are past knowledge and the duration of UC, which may impact the prognosis and management of this condition and potentially alter the study results since patients learn to self-manage their condition over time. Future work should account for any differences in additional confounding parameters, including past knowledge and length of disease between their experimental groups.

## Conclusions

The present study examined the effect of SP supplementation on disease activity, health-related quality of life, serum antioxidant status, and PTX-3 status in patients with UC. Our findings indicate that TAC and stool frequency improved after SP supplementation in this population. In addition, SP supplementation did not change disease activity parameters, PTX-3 levels and ESR. Our findings suggest that SP supplementation may be effective as an adjuvant treatment for managing patients with UC. Therefore, larger trials with longer interventions periods are required to draw more precise conclusions.

## Data Availability

The datasets generated and/or analysed during the current study are not publicly available due but are available from the corresponding author on reasonable request.
